# Supplementary material for: Critical weaknesses in shielding strategies for COVID-19
Source: PLOS Glob Public Health. 2022 Apr 26;2(4):e0000298. doi: 10.1371/journal.pgph.0000298 (PMC10021285; doi:10.1371/journal.pgph.0000298)
Supplement: S1 Text — (DOCX) [file pgph.0000298.s001.docx]

**Supplementary Material: Critical weaknesses in shielding strategies for COVID-19**

**Authors:** Cameron A. Smith^1^, Christian A. Yates^1^†, Ben Ashby^1,2^†*

**Affiliations:**

^1^Department of Mathematical Sciences, University of Bath; Bath, BA2 7AY, UK.
^2^Milner Centre for Evolution, University of Bath; Bath, BA2 7AY, UK.

*Corresponding author. Email: bna24@bath.ac.uk.

†Equal contribution

Keywords: non-pharmaceutical interventions, herd immunity, shielding, focused protection, SARS-CoV-2, COVID-19

# §A: Sensitivity analysis

In this section, we present additional results which demonstrate that the qualitative results presented in the main text are robust to reasonable variations in parameters. Specifically, we vary the effectiveness of shielding (Fig. A) and investigate the effect of changing the reduction in contact by lower-risk individuals during the shielding phase (Fig. B and Fig. C), the basic reproductive number (Fig. D and Fig. E) and the proportion of contacts within LTC facilities (Fig. F and Fig. G). Furthermore, we verify that our results are robust when varying the incubation period (Fig. H and Fig. I), the infectious period (Fig. J and Fig. K), the force of external infection (Fig. L and Fig. M), and robust to variation in LTC facility size (Fig. N).

We begin by varying the effectiveness of shielding in the imperfect shielding scenario by 10% either side of the baseline of 80% employed in the main text (Fig. A). Unsurprisingly, decreasing the effectiveness of the shielding increases the number of deaths in the higher-risk subpopulations, while increasing the effectiveness of shielding has the opposite effect. The qualitative epidemiological dynamics results are broadly similar, however.

We next vary the contact reduction among the lower-risk population, from the 50% employed in the main text, to a 40% and 60% reduction in both the perfect shielding (Fig. B) and imperfect shielding (Fig. C) cases. In the perfect shielding case, a greater reduction in the contact rate among lower-risk members of the population during shielding is associated with a longer shielding phase (420 days on average for 60% reduction, 300 days on average for 50% reduction, 200 days on average for 40% reduction) and a larger second wave, particularly in the higher-risk in the community. We see the same broad pattern when considering the imperfect shielding scenario (Fig. C).

Next, we investigate the effects of varying the basic reproduction number from $R_{0}=3$ (employed in the main text scenarios) to $R_{0}=2.5$ or $R_{0}=3.5$. We conduct these simulations on both the imperfect shielding (IS; Fig. D) and imperfect shielding with reduced contact (IS + RC; Fig. E) scenarios, as there is very little effect on the results for perfect shielding. For the IS scenario (Fig. D), we see an increase in deaths and infections as the basic reproduction number increases, however the qualitative behaviour remains unchanged. When reduced contact among lower-risk individuals is included (Fig. E), we see that increasing $R_{0}$ results in a larger and shorter first wave, and a smaller second wave. When $R_{0}$ is decreased, the first wave is much longer with very few cases, but a notable increase in cases during the second wave.

Now we vary the value of $\lambda$, the proportion of contacts that an LTC resident has within their LTC facility. Again, we conduct this analysis on both the imperfect shielding (IS; Fig. F) and imperfect shielding with reduced contact (IS + RC; Fig. G) scenarios. In both cases there are no qualitative effects on the results.

In the main text, we fix the incubation and infectious periods at $1/\sigma=2$ and $1/\Gamma=5$ respectively. In Figs. H and I we alter the incubation period and in Figs. J and K we alter the infectious period. In each of these plots, we see no qualitative differences in our results. As expected, the numbers of deaths are unaltered, with only the infection profiles changing to become either narrower yet taller, or wider and shorter. Similarly, we see no qualitative change in our results when varying the external force of infection with either imperfect shielding (Fig. L) or perfect shielding (Fig. M). We also assess whether the distribution of care home sizes affects the outcome by replacing small, medium and large LTC facilities with 180 identical LTCs of size 40. This yields the same number of LTC residents, only they are now distributed into equally sized homes. This redistribution does not alter our conclusions (Fig. N).

# §B: Splitting the infection class into symptomatic and asymptomatic classes

In the main text, in order to ensure that the model remains as simple as possible, we consider just a single infectious class, which combines symptomatic and asymptomatic individuals . Our transmission and IFR parameters reflect this choice and represent ensemble averages over symptomatic and asymptomatic infections. In this section we demonstrate that explicitly separating the infection class into symptomatic and asymptomatic classes does not qualitatively affect our conclusions.

We define $I_{S,i}\left( t \right)$ and $I_{A,i}\left( t \right)$ to be the number of symptomatic and asymptomatic individuals in the $i$^th^ subpopulation at time $t$. We assume that a proportion $\kappa$ of infected individuals become symptomatic, only those who are symptomatic die from the disease, and that symptomatic and asymptomatic individuals may differ in their transmissibility and IFRs (indicated by tildes). Then we can write analogous transition probabilities to those in the main text (equations (7)) with a new state variable for the $i$^th^ subpopulation at time $t$, ${\underline{\tilde{Y}}}_{i}^{t}=\left( S_{i}, E_{i}, I_{S,i}, I_{A,i}, R_{i}D_{i} \right)$:

$$\mathbb{P}\left( {\underline{\tilde{Y}}}_{i}^{t+\delta t}-{\underline{\tilde{Y}}}_{i}^{t}=\left( -1,1,0,0,0,0 \right) \right)=\left( \eta_{i}\left( t \right)S_{i}+\sum_{j} \frac{Q_{ij}S_{i}}{N_{j}}\left( \tilde{\beta}_{ij}^{S}I_{S,j}+ \tilde{\beta}_{ij}^{A}I_{A,j} \right) \right)\delta t,$$

$$\mathbb{P}\left( {\underline{\tilde{Y}}}_{i}^{t+\delta t}-{\underline{\tilde{Y}}}_{i}^{t}=\left( 0,-1,1,0,0,0 \right) \right)=\sigma\kappa E_{i}\delta t,$$

$$\mathbb{P}\left( {\underline{\tilde{Y}}}_{i}^{t+\delta t}-{\underline{\tilde{Y}}}_{i}^{t}=\left( 0,-1,0,1,0,0 \right) \right)=\sigma\left( 1-\kappa\right)E_{i}\delta t,$$

$$\mathbb{P}\left( {\underline{\tilde{Y}}}_{i}^{t+\delta t}-{\underline{\tilde{Y}}}_{i}^{t}=\left( 0,0,-1,0,1,0 \right) \right)=\Gamma\left( 1-\tilde{\alpha}_{i} \right)I_{S,i}\delta t,$$

$$\mathbb{P}\left( {\underline{\tilde{Y}}}_{i}^{t+\delta t}-{\underline{\tilde{Y}}}_{i}^{t}=\left( 0,0,-1,0,0,1 \right) \right)=\Gamma\tilde{\alpha}_{i}I_{S,i}\delta t,$$

$$\mathbb{P}\left( {\underline{\tilde{Y}}}_{i}^{t+\delta t}-{\underline{\tilde{Y}}}_{i}^{t}=\left( 0,0,0,-1,1,0 \right) \right)=\Gamma I_{A,i}\delta t.$$

The transmission rates $\tilde{\beta}_{ij}^{S}$ and $\tilde{\beta}_{ij}^{A}$ are similar, but with the symptomatic case being larger than the asymptomatic case. Note that the transmission parameter is constructed from the probability of transmission per contact, the average number of contacts a person has per unit time, the contact matrix $p_{ij}$ and the shielding matrix $Q_{ij}$. We assume that in both symptomatic and asymptomatic cases, the contact and shielding matrices remain the same as these are functions of the subpopulation to which an individual belongs. We assume that symptomatic individuals shed more virus and so have a higher probability of onward transmission per contact, but have a lower average number of contacts per unit time as they are more likely to self-isolate due to symptoms. We run simulations for each of the shielding scenarios with no modifications, setting $\tilde{\beta}_{ij}^{S}=1.1\beta_{ij}$ and $\tilde{\beta}_{ij}^{A}=0.8\beta_{ij}$, where $\beta_{ij}$ is the same transmission rate as in the main text. Further, we set $\kappa=2/3$, and $\tilde{\alpha}_{i}=\alpha_{i}/\kappa$ for symptomatic individuals, with $\tilde{\alpha}_{i}=0$ for asymptomatic individuals. The results from this can be observed in Fig. O and show no qualitative difference to those in the main text Fig. 2.

# §C: The effective reproductive number

In order to estimate the effective reproductive number, $R_{e}(t)$, we multiply the basic reproductive number $R_{0}$ calculated as in §2.2 in the main text by the proportion of susceptible individuals at time $t$:

$$R_{e}\left( t \right)=\frac{R_{0}\left( t \right)S\left( t \right)}{N}$$

Note that we have explicitly written $R_{0}$ as a function of time here because it depends on whether we are in the shielding period. If we are within the shielding period, the value corresponds to the largest root of the characteristic equation (16a) with the appropriate shielding parameter values. When we are outside of the shielding period, the value of $R_{0}$ is $\beta_{0}r/\Gamma$. We show these plots in time for each of the main text scenarios in Fig. S16. As expected, reducing the contact of lower-risk individuals during shielding results in the effective reproductive number increasing above one when shielding ends. Waning immunity also sees the effective reproductive number oscillate around 1 as the pool of susceptibles shrinks and grows with the waxing and waning of each epidemic wave. In the other two cases (no modifiers and external infection), the overall effective reproductive number does not exceed one once shielding ends. Again, this is to be expected as we have reached herd immunity, but this only confers indirect protection, so those in the lesser exposed subpopulations are still vulnerable to local outbreaks.


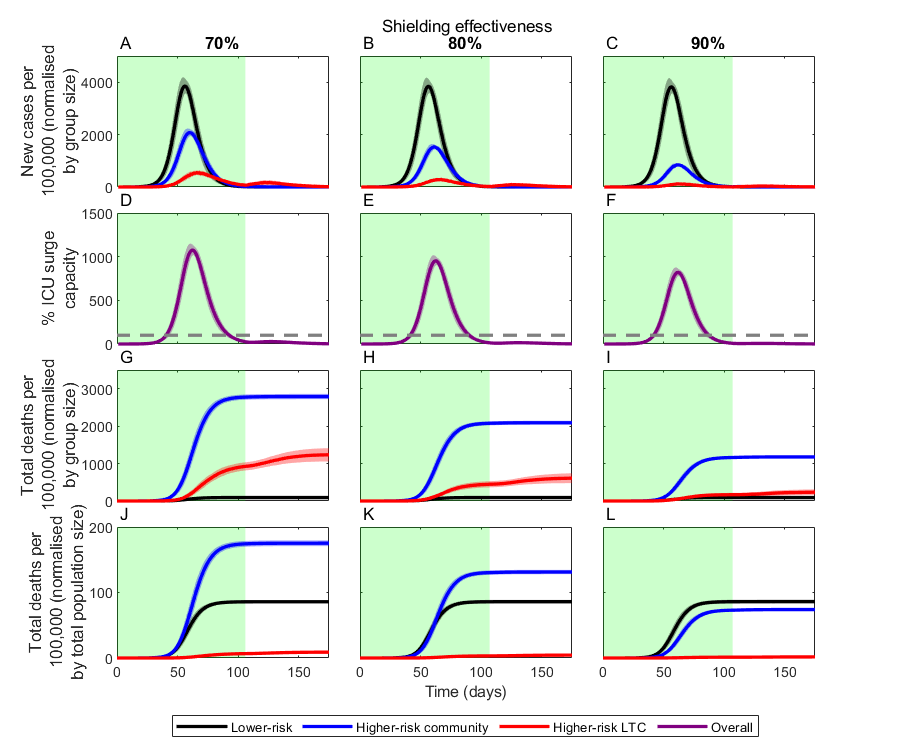


Fig. A: Effectiveness of imperfect shielding. Baseline figure (reproduced from Fig. 1 in the main text) is in the central column (80% effective), with a 10% difference on either side (70% for column 1 and 90% for column 3). All colours and descriptions are the same as Fig. 2 of the main text. Lines correspond to means for groups at: lower-risk (black), higher-risk in the community (blue) and in LTC facilities (red), with shading indicating ± 1 SD. Green shading indicates the shielding phase.


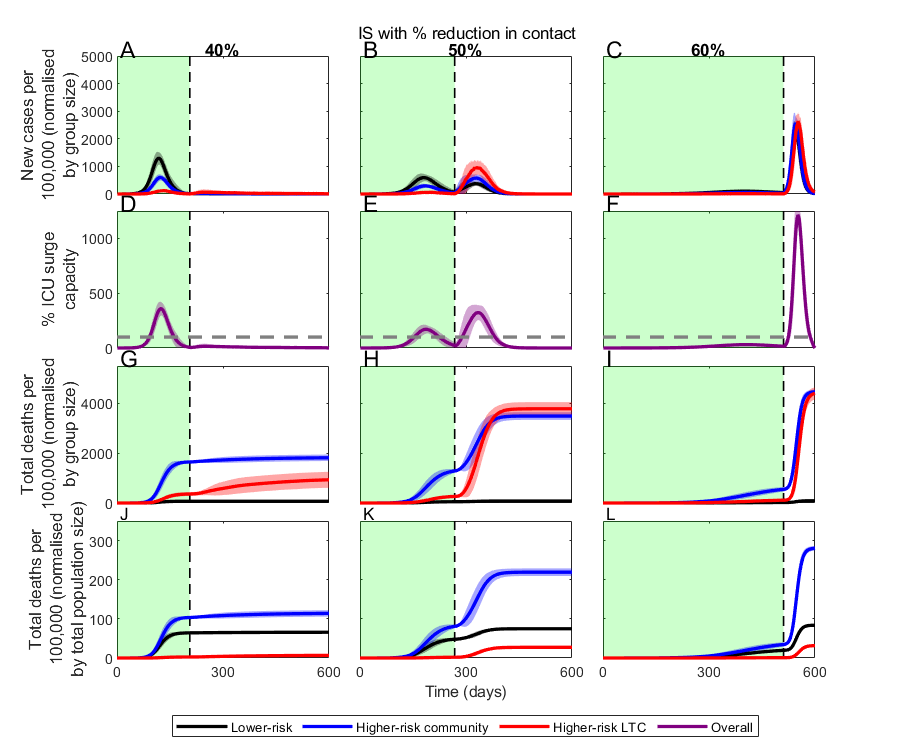


Fig. B: The effects of altering the reduction in contacts among lower-risk individuals during the shielding phase under imperfect shielding. The case from the main text (50% reduction, reproduced from Fig. 3) is shown in the second column, with a change of -/+ 10% either side in columns 1 and 3 respectively. All colours and descriptions are the same as Fig. 2 of the main text. Lines correspond to means for groups at: lower-risk (black), higher-risk in the community (blue) and in LTC facilities (red), with shading indicating ± 1 SD. Green shading indicates the shielding phase.


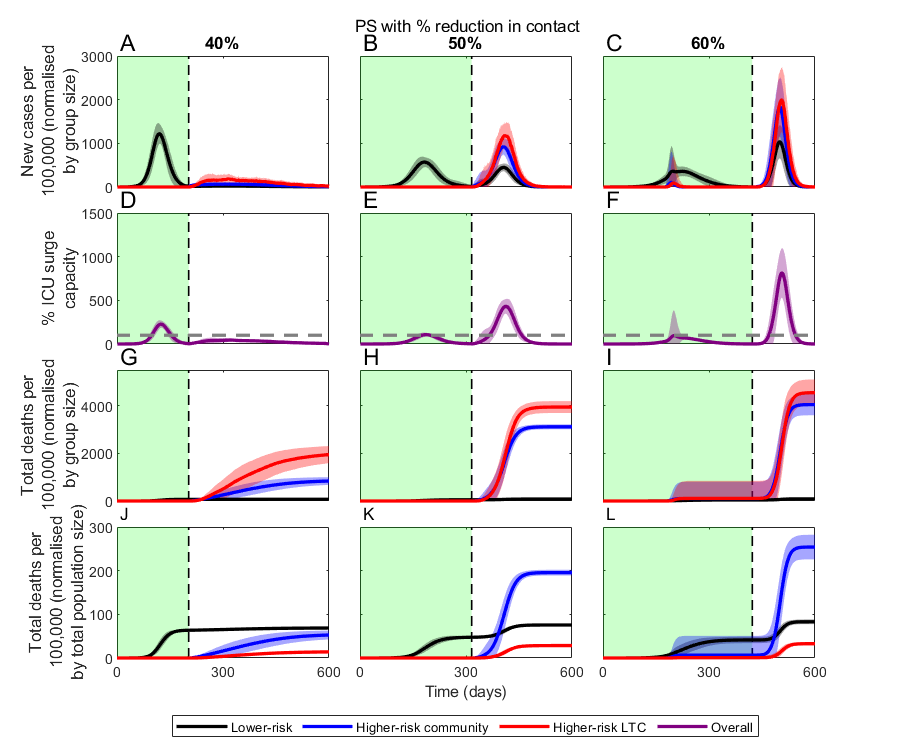


Fig. C: The effects of altering the reduction in contacts among lower-risk individuals during the shielding phase under perfect shielding. The case from the main text (50% reduction, reproduced from Fig. 3) is shown in the second column, with a change of -/+ 10% either side in columns 1 and 3 respectively. All colours and descriptions are the same as Fig. 2 of the main text. Lines correspond to means for groups at: lower-risk (black), higher-risk in the community (blue) and in LTC facilities (red), with shading indicating ± 1 SD. Green shading indicates the shielding phase.


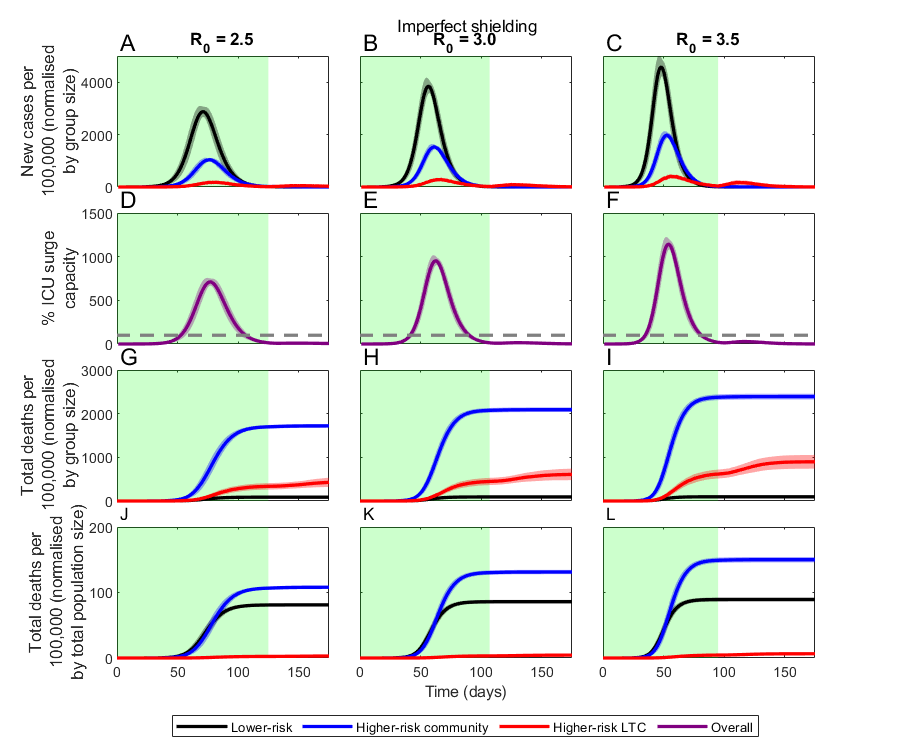


Fig. D: Altering $R_{0}$ under the imperfect shielding scenario. The second column is reproduced from Fig. 1. Column 1 represents a reduction in R_0_  to 2.5 and column 3 an increase to 3.5. All colours and descriptions are the same as Fig. 2 of the main text. Lines correspond to means for groups at: lower-risk (black), higher-risk in the community (blue) and in LTC facilities (red), with shading indicating ± 1 SD. Green shading indicates the shielding phase.


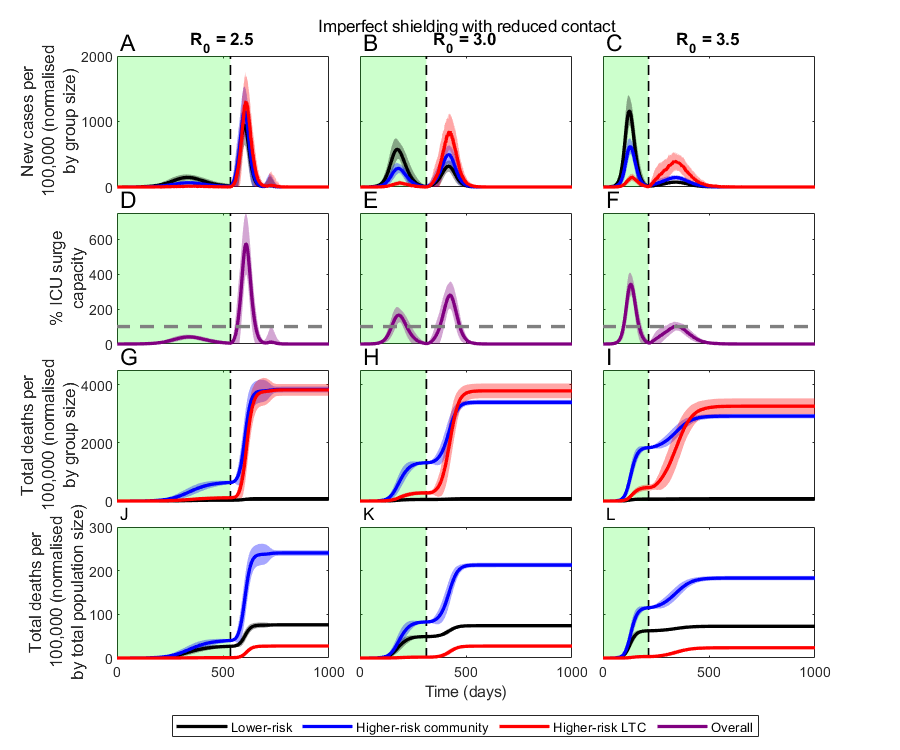


Fig. E: Altering $R_{0}$ under the imperfect shielding scenario with reduced contact. The second column is reproduced from Fig. 4. Column 1 represents a reduction in R_0_  to 2.5 and column 3 an increase to 3.5. All colours and descriptions are the same as Fig. 2 of the main text. Lines correspond to means for groups at: lower-risk (black), higher-risk in the community (blue) and in LTC facilities (red), with shading indicating ± 1 SD. Green shading indicates the shielding phase. NS: no shielding; IS: imperfect shielding; PS: perfect shielding.


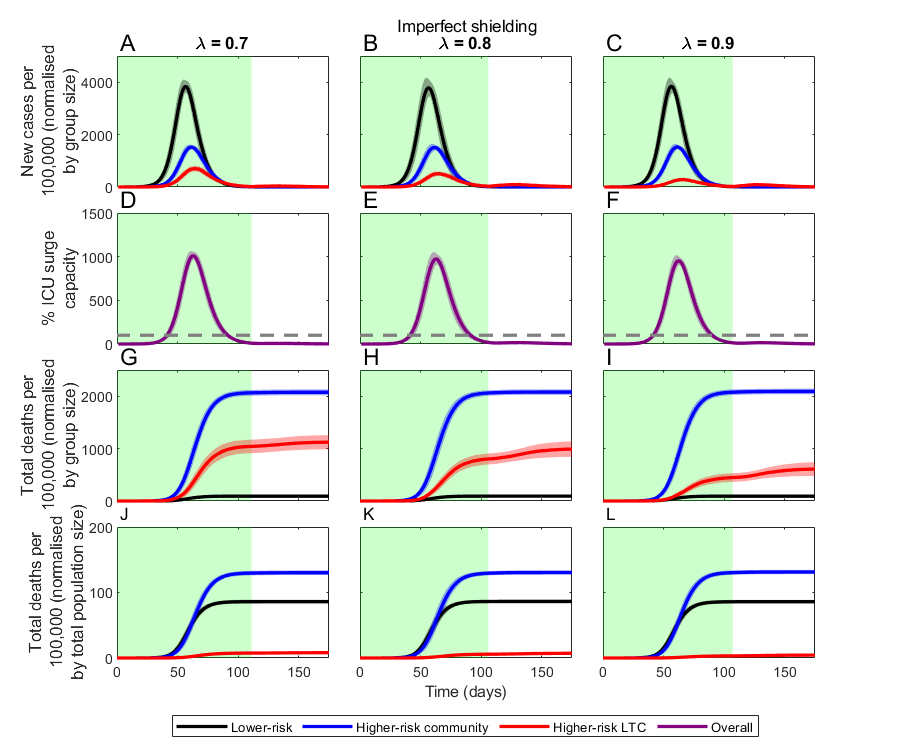


Fig. F: Altering $\lambda$ under the imperfect shielding scenario. The third column is reproduced from Fig. 1. Column 1 represents a reduction in $\lambda$ to 0.7 and column 2 an decrease to 0.8. All colours and descriptions are the same as Fig. 2 of the main text. Lines correspond to means for groups at: lower-risk (black), higher-risk in the community (blue) and in LTC facilities (red), with shading indicating ± 1 SD. Green shading indicates the shielding phase.


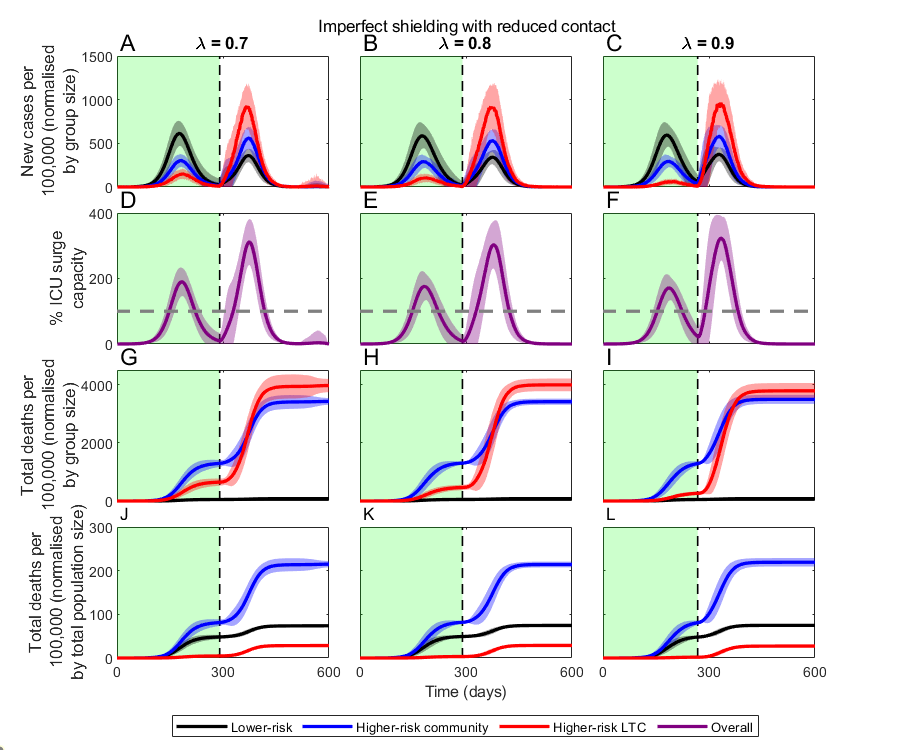


Fig. G: Altering $\lambda$ under the imperfect shielding scenario with reduced contact. The third column is reproduced from Fig. 4. Column 1 represents a reduction in $\lambda$ to 0.7 and column 2 an decrease to 0.8. All colours and descriptions are the same as Fig. 2 of the main text. Lines correspond to means for groups at: lower-risk (black), higher-risk in the community (blue) and in LTC facilities (red), with shading indicating ± 1 SD. Green shading indicates the shielding phase.


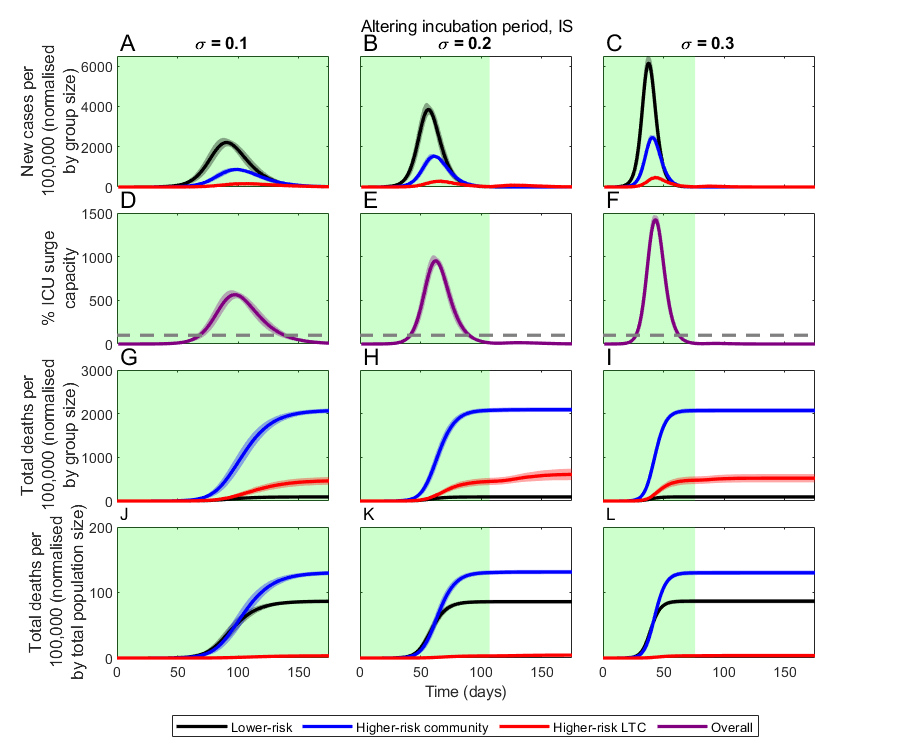


Fig. H: Altering the incubation period $1/\sigma$ under the imperfect shielding scenario. The second column is reproduced from Fig. 2. Column 1 represents a reduction in $\sigma$ to 0.1 and column 3 an increase to 0.3. All colours and descriptions are the same as Fig. 2 of the main text. Lines correspond to means for groups at: lower-risk (black), higher-risk in the community (blue) and in LTC facilities (red), with shading indicating ± 1 SD. Green shading indicates the shielding phase.


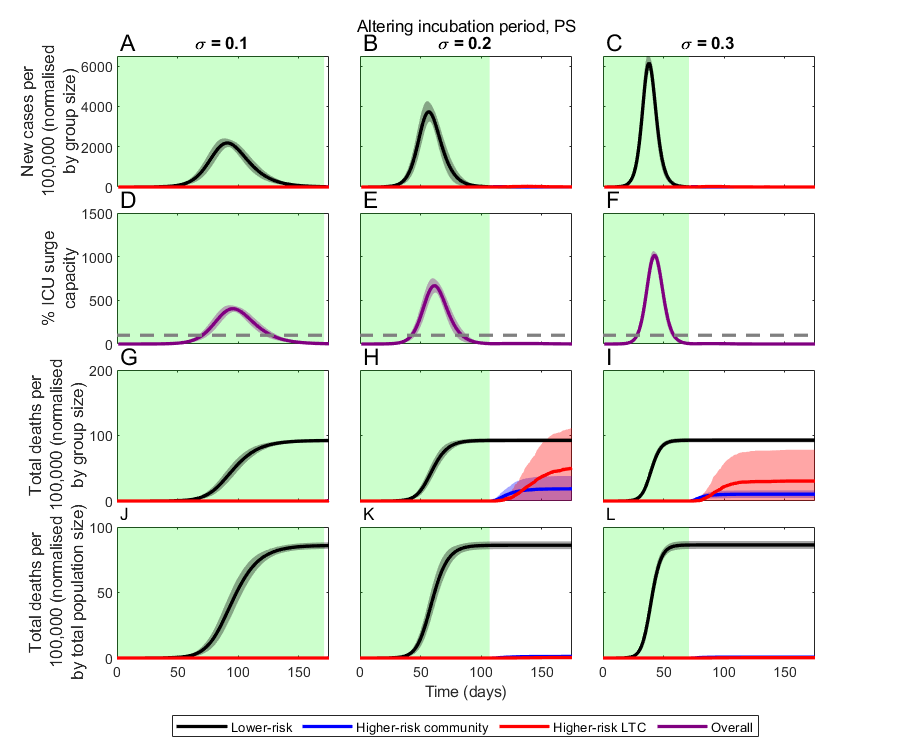


Fig. I: Altering the incubation period $1/\sigma$ under the perfect shielding scenario. The second column is reproduced from Fig. 2. Column 1 represents a reduction in $\sigma$ to 0.1 and column 3 an increase to 0.3. All colours and descriptions are the same as Fig. 2 of the main text. Lines correspond to means for groups at: lower-risk (black), higher-risk in the community (blue) and in LTC facilities (red), with shading indicating ± 1 SD. Green shading indicates the shielding phase.


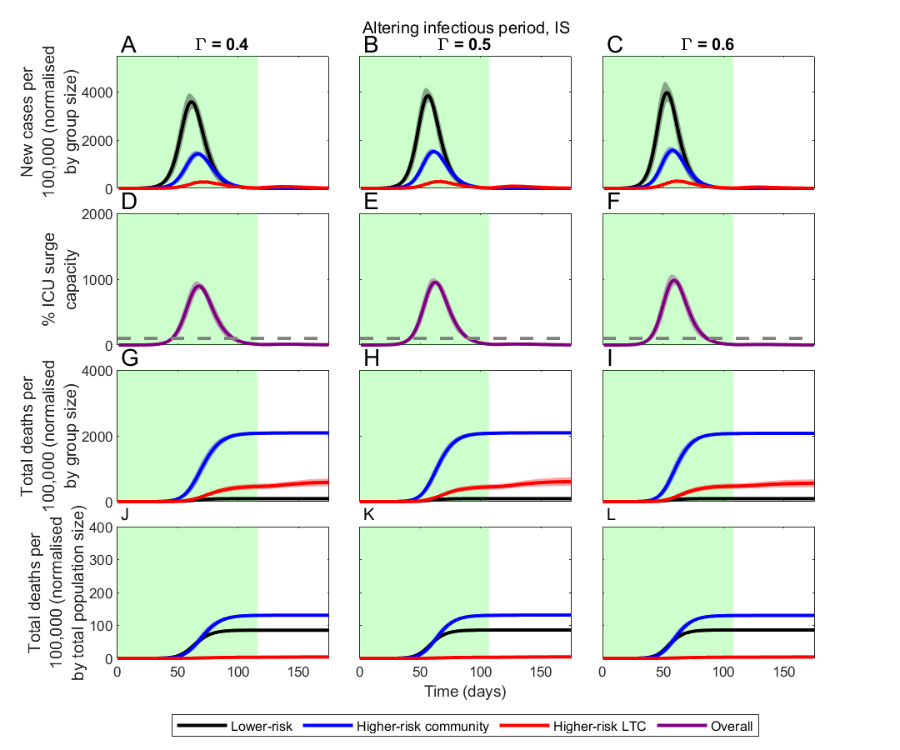


Fig. J: Altering the infectious period $1/\Gamma$ under the imperfect shielding scenario. The second column is reproduced from Fig. 2. Column 1 represents a reduction in $\Gamma$ to 0.4 and column 3 an increase to 0.6. All colours and descriptions are the same as Fig. 2 of the main text. Lines correspond to means for groups at: lower-risk (black), higher-risk in the community (blue) and in LTC facilities (red), with shading indicating ± 1 SD. Green shading indicates the shielding phase.


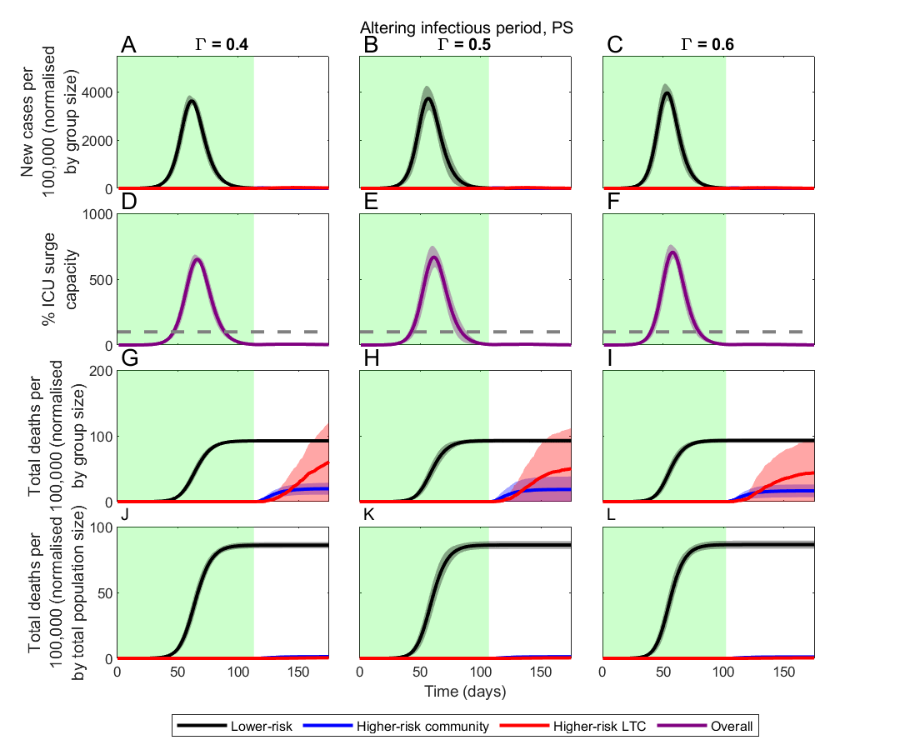


Fig. K: Altering the infectious period $1/\Gamma$ under the perfect shielding scenario. The second column is reproduced from Fig. 2. Column 1 represents a reduction in $\Gamma$ to 0.4 and column 3 an increase to 0.6. All colours and descriptions are the same as Fig. 2 of the main text. Lines correspond to means for groups at: lower-risk (black), higher-risk in the community (blue) and in LTC facilities (red), with shading indicating ± 1 SD. Green shading indicates the shielding phase.


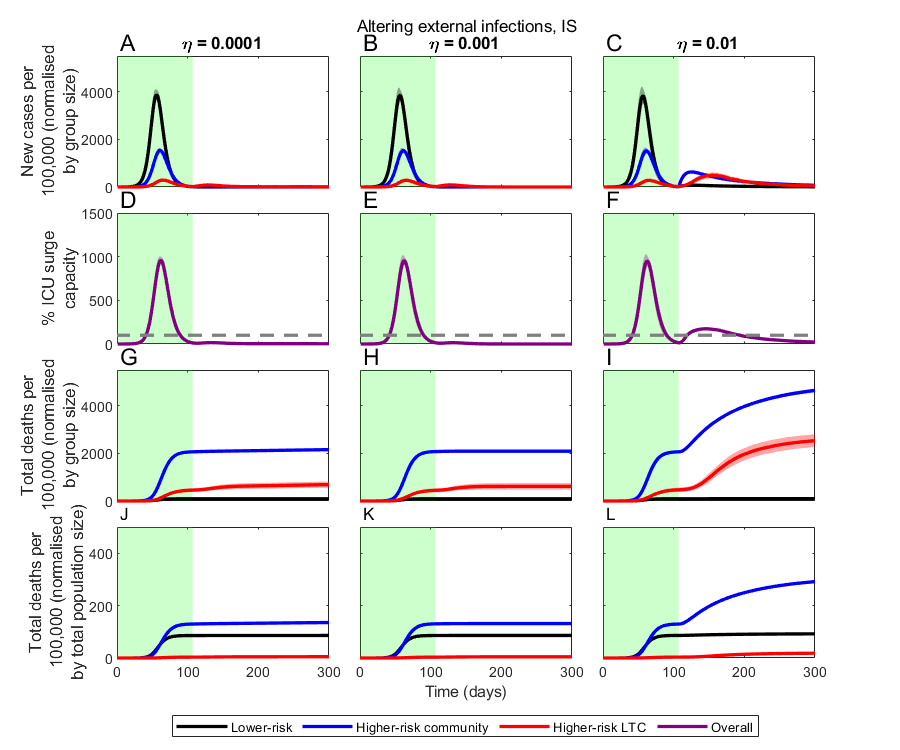


Fig. L: Altering the external force of infection, $\eta$, under the imperfect shielding scenario. The second column is reproduced from Fig. 5. Column 1 represents a reduction in $\eta$ to 0.0001 and column 3 an increase to 0.01. All colours and descriptions are the same as Fig. 2 of the main text. Lines correspond to means for groups at: lower-risk (black), higher-risk in the community (blue) and in LTC facilities (red), with shading indicating ± 1 SD. Green shading indicates the shielding phase.


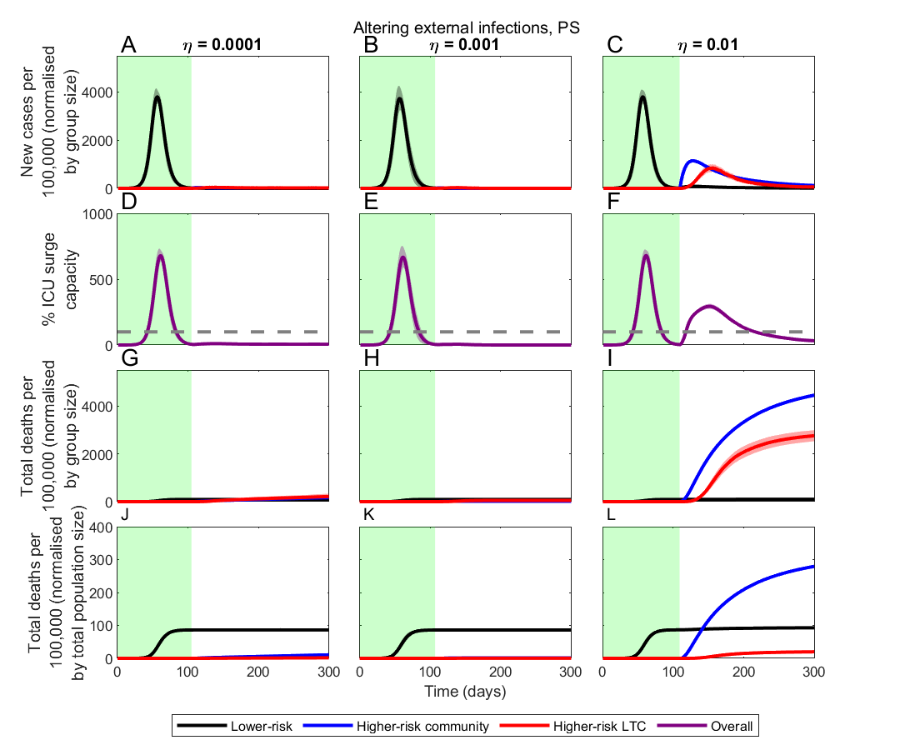


Fig. M: Altering the external force of infection, $\eta$, under the perfect shielding scenario. The second column is reproduced from Fig. 5. Column 1 represents a reduction in $\eta$ to 0.0001 and column 3 an increase to 0.01. All colours and descriptions are the same as Fig. 2 of the main text. Lines correspond to means for groups at: lower-risk (black), higher-risk in the community (blue) and in LTC facilities (red), with shading indicating ± 1 SD. Green shading indicates the shielding phase.


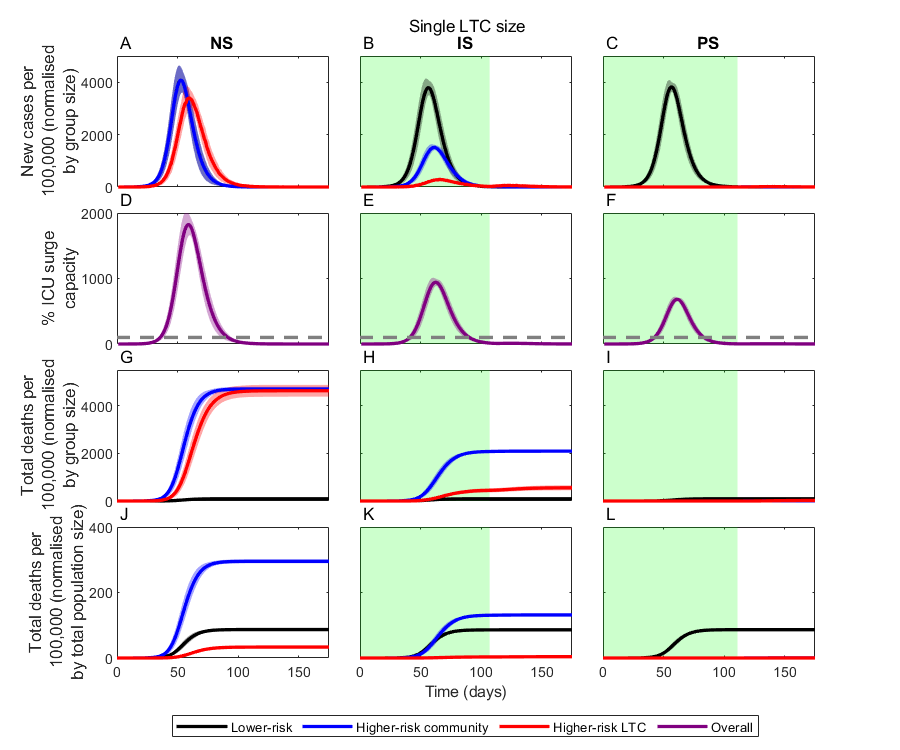


Fig. N: Results with a single LTC facility size (180 LTCs each with 40 residents), in contrast to Fig. 2 of the main text where we have small, medium and large LTC facilities. All colours and descriptions are the same as Fig. 2 of the main text. Lines correspond to means for groups at: lower-risk (black), higher-risk in the community (blue) and in LTC facilities (red), with shading indicating ± 1 SD. Green shading indicates the shielding phase. NS: no shielding; IS: imperfect shielding; PS: perfect shielding.


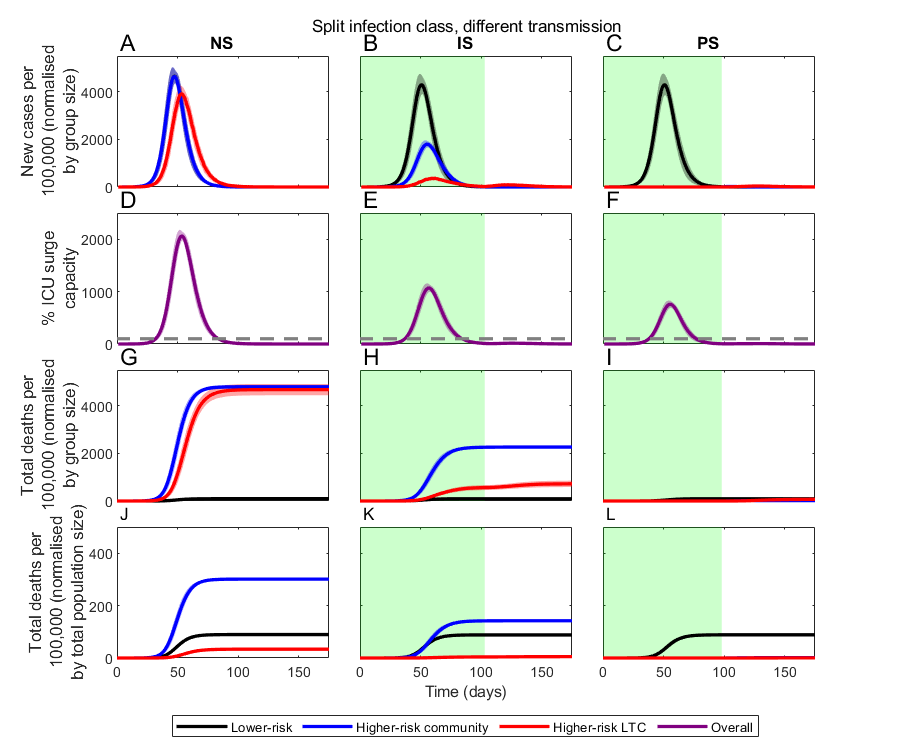


Fig. O: Results with separate asymptomatic and symptomatic classes. All colours and descriptions are the same as Fig. 2 of the main text. Lines correspond to means for groups at: lower-risk (black), higher-risk in the community (blue) and in LTC facilities (red), with shading indicating ± 1 SD. Green shading indicates the shielding phase. NS: no shielding; IS: imperfect shielding; PS: perfect shielding.


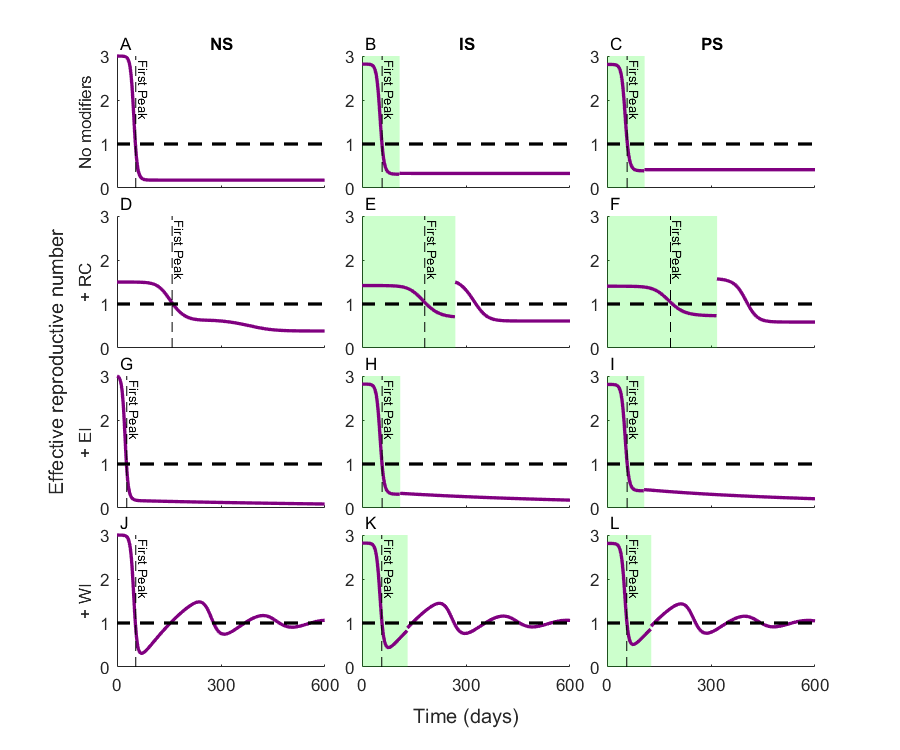


Fig. P: The effective reproductive number for each of the scenarios described in the main text. The first column represents no shielding (NS), the middle column imperfect shielding (IS) and the third column perfect shielding (PS). Row 1 corresponds to no additional modifiers, row 2 includes reduced contact (RC) at 50%, row 3 adds external infections (EI) after shielding and row 4 has waning immunity (WI). The black horizontal dashed line denotes the effective reproductive number equalling one, and the grey horizontal line is the time at which the first peak occurs in the incidence curves, coinciding with the point that the effective reproductive number hits 1.

| Scenario | Shielding parameter | | | | | |
| --- | --- | --- | --- | --- | --- | --- |
|  | $q_{1}^{s}$ | $q_{2}^{S}$ | $q_{3}^{S}$ | $q_{4}^{S}$ | $q_{5}^{S}$ | $q_{6}^{s}$ |
| NS (+WI) | 1 | 1 | 1 | 1 | 1 | 1 |
| IS (+WI) | 1 | 0.2 | 0.2 | 0.2 | 0.2 | 0 |
| PS (+WI) | 1 | 0 | 0 | 0 | 0 | 0 |
| NS + RC | 0.5 | 0.5 | 0.5 | 0.5 | 0.5 | 0.5 |
| IS + RC | 0.5 | 0.2 | 0.2 | 0.2 | 0.2 | 0 |
| PS + RC | 0.5 | 0 | 0 | 0 | 0 | 0 |
| NS + EI | 1 | 1 | 1 | 1 | 1 | 1 |
| IS + EI | 1 | 0.2 | 0.2 | 0.2 | 0.2 | 0 |
| PS + EI | 1 | 0 | 0 | 0 | 0 | 0 |

Table A: Parameter values for the different shielding scenarios and modifiers.

| **Scenario** | **Deaths per 100,000 (**$\boldsymbol{\pm}$ **1 standard deviation)** | | | |
| --- | --- | --- | --- | --- |
|  | **Lower-risk** | **Higher-risk (community)** | **Higher-risk**  **(LTC residents)** | **Overall** |
| **NS**  (Fig. 2, col. 1) | 93.7  (90.3, 97.1) | 4702.1  (4615.9, 4788.3) | 4532.6  (4280.0, 4785.2) | 415.1  (408.5, 421.6) |
| **IS**  (Fig. 2, col. 2) | 92.4  (89.4, 95.5) | 2090.9  (2038.0, 2143.8) | 613.6  (478.4, 748.8) | 221.7  (217.8, 225.5) |
| **PS**  (Fig. 2, col. 3) | 92.5  (89.4, 95.7) | 18.6  (0.0, 38.3) | 54.3  (0.0, 123.3) | 87.6  (84.2, 91.1) |
| **NS + RC**  (Fig. 3, col. 1) | 74.2  (71.4, 77.0) | 3712.8  (3633.0, 3792.6) | 3336.5  (3114.1, 3558.8) | 326.2  (319.8, 332.5) |
| **IS + RC**  (Fig. 3, col. 2) | 80.3  (77.4, 83.3) | 3491.5  (3337.7, 3645.3) | 3783.7  (3516.2, 4051.2) | 321.2  (309.7, 332.7) |
| **PS + RC**  (Fig. 3, col. 3) | 81.2  (78.2, 84.3) | 3114.4  (3010.8, 3218.0) | 3944.2  (3695.7, 4192.6) | 299.5  (292.0, 307.1) |

Table B: Deaths per 100,000 (normalised by group) for the main text scenarios. The darker the colour in a column, the higher the number of deaths.

| Reference | 2011 Census | Report 9 (1) (* indicates interpolated data) | | |
| --- | --- | --- | --- | --- |
| Age group | Population (%) | Symptomatic requiring hospital treatment (%) | Hospitalised requiring ICU support (%) | Symptomatic requiring ICU support (%) |
| 0-9 | 11.8 | 0.1 | 5.0 | 0.005 |
| 10-19 | 12.1 | 0.3 | 5.0 | 0.015 |
| 20-29 | 13.6 | 1.2 | 5.0 | 0.060 |
| 30-39 | 13.1 | 3.2 | 5.0 | 0.160 |
| 40-49 | 14.6 | 4.9 | 6.3 | 0.309 |
| 50-59 | 12.2 | 10.2 | 12.2 | 1.244 |
| 60-64 | 6.0 | 15.0* | 23.6* | 3.722* |
| 65-69 | 4.8 | 18.5* | 31.4* | 6.036* |
| 70-79 | 7.1 | 24.3 | 43.2 | 10.498 |
| 80+ | 4.7 | 27.4 | 70.9 | 19.356 |

Table C: The values used for the hospitalisation calculations. The population breakdown is from the 2011 census, while the data in the final three columns is taken from [1]. An asterisk denotes data that has been interpolated.

# References

1. Ferguson NM, Laydon D, Nedjati-Gilani G, Imai N, Ainslie K, Baguelin M, et al. Impact of non-pharmaceutical interventions (NPIs) to reduce COVID-19 mortality and healthcare demand. 2020; Available from: https://doi.org/10.25561/77482
