# Supplementary material for: Critical weaknesses in shielding strategies for COVID-19
Source: PLOS Glob Public Health. 2022 Apr 26;2(4):e0000298. doi: 10.1371/journal.pgph.0000298 (PMC10021285; doi:10.1371/journal.pgph.0000298)
Supplement: Attachment — Submitted filename: Referee_comments.docx [file pgph.0000298.s002.docx]

**Department of Mathematical Sciences, University of Bath**

Claverton Down, Bath BA2 7AY, UK

February 9, 2022

Dear Editor,

Thank you for the reviews for our manuscript: “Critical weaknesses in shielding strategies for COVID-19”. We have carefully considered the reviewers’ comments and have revised our manuscript accordingly. In particular, we have carried out additional sensitivity analysis, added new results, and have included clarifications throughout the text in response to these comments. As the purpose of our study is to illustrate critical problems with shielding strategies, we have declined to make certain suggested changes that would greatly increase the complexity of the model without likely altering our key conclusions. Please find our detailed responses below, in which comments by reviewers are *italicised* and our responses are in regular text underneath. Line numbers refer to the new version of the manuscript “herd_immunity_main_update.doc”

Reviewer 1:

1. *Justification/referencing is needed to clarify the choice of population sizes for small, medium and large LTC facilities (lines 86-90).*

Anecdotally, we know that there is considerable variation in the size of LTC facilities in England, from very small facilities with fewer than ten residents to very large facilities with hundreds of residents. We felt it appropriate to include heterogeneity in LTC facility sizes, but data on LTC facility sizes are scarce. According to a study by Knight Frank, as of April 2019 there were around 12,250 LTC facilities in the UK with 477,100 beds, giving an average of 39 beds per facility. We therefore chose relatively small, medium, and large care home sizes such that we have a similar average number of beds per facility (37.5) to the national average, with individuals spread evenly between facilities of different sizes. While this is obviously a simplification, it allows us to qualitatively capture some of the variation that exists in real LTC facility sizes. Sensitivity analysis reveals that our conclusions are robust when we instead assume that all LTC facilities are of the same size (Fig. S14). The following sentence has been added to lines 96-97 to justify our assumptions about LTC facility sizes: “This gives an average of 37.5 residents per LTC facility, which is close to the UK average 39 (16).”

2. *There is also a need to describe the parameterisation for the transmission pressure from external infections,* $\eta_{i}(t)$*. This is important considering that Fig. 4 is a key result for the paper (line 119).*

The purpose of this figure is to demonstrate that if there is a sufficient force of external infections, deaths will rise substantially after shielding ends because herd immunity only confers indirect protection to the vulnerable. The choice of this parameter is therefore arbitrary so long as it is large enough to cause infections without being unrealistically high such that it dominates the dynamics. We have now placed more emphasis on the qualitative results relating to external infections. We have also conducted sensitivity analysis on this parameter in Figs. S12-S13 to demonstrate that this effect is robust as the external force of infection varies. Intuitively, as the external force of infection increases, deaths in the post-shielding phase will tend to be higher.

3. *The authors need to explicitly describe parameters* $pop_{l}$ *and* $ICU_{l}$ *in section 2.3. It is not clear what values in table S3 the equation is referring to.*

This has now been added on lines 237-238: “Also, $pop_{\mathcal{l}}$ is the proportion of the population in the age category $\mathcal{l}$, and $ICU_{\mathcal{l}}$ is the probability that a symptomatic individual in age category $\mathcal{l}$ is admitted to ICU.”

4. *The authors have placed significant emphasis on discussing their results in the context of herd immunity (e.g lines 252-259, 267-268). However, little quantitative exploration of the dynamics of herd immunity or herd immunity thresholds (HIT) has been performed in this study. Supplementary analyses which demonstrate how certain scenarios fail to reach HIT due to insufficient depletion of susceptibles, or reach HIT but still result in excessive number of deaths would be useful to support the statements made in the results section.*

We have added an investigation of the effective reproductive number to the supplementary material, which can be found in Section S3 and Fig. S16.

5. *Hospitalisations exceeding ICU capacity is referenced in the results and plotted on figures, but there is no description in the main text what this ICU capacity or threshold value is (line 224-225, 233-234, 250, 279-280).*

We have now described this on lines 239-240 “The ICU capacity for the UK is approximately 8 ICU beds per 100,000 people (18).”.

6. *The authors do not describe the impact of external infections in Fig. 4 sufficiently before contextualising the figure with regards to herd immunity (lines 254-262). The increase in total deaths per 100,000 for IS + EI and PS + EI scenarios is an important result and deserves further explanation. It would also be of interest to conduct an additional sensitivity analysis ranging the extent of* $\eta_{i}(t)$ *and how this affects Fig. 4.*

We have reordered our results to first focus on imperfect shielding, then reduced contact by lower-risk individuals, and finally on the fact that herd immunity only confers indirect protection and is temporary. The impact of external infections (EI) now comes in the final section (Fig. 5) and is grouped with waning immunity (WI; Fig. 6), as both demonstrate problems with relying on herd immunity to protect the vulnerable. Unlike imperfect shielding and reduced contact by lower-risk individuals, which both result in epidemic burnout, EI and WI both lead to a continual increase in deaths and so do not have a comparable endpoint to the previous scenarios, which makes the quantitative results less meaningful. We therefore now focus on the qualitative effects of EI and WI. As described above in response to comment 2, we have added sensitivity analysis for EI to the supplementary material (Fig. S12-13). We have also added additional explanations for EI and WI to lines 324-329: “In our simulations, we see that an external force of infection leads to a steady increase in deaths after shielding is lifted (Fig. 5), both among higher-risk individuals in the community despite herd-immunity being achieved, and among clusters of higher-risk individuals in LTC facilities in which herd immunity has not been achieved locally. Similarly, waning immunity leads to a resurgence in cases following the relaxation of shielding, leading to a substantial increase in deaths among those at greatest risk (Fig. 6).”

7. *It is not clear what the three critical weaknesses are when the authors refer to "A third critical weakness…" (line 267). These weaknesses need to be made more explicit in the text.*

We now identify the first critical weakness as imperfect shielding (line 274), the second as behaviour change among lower-risk individuals (line 288), and the third as herd immunity only conferring indirect protection and being temporary (line 309).

8. *Sensitivity analyses should be referenced within the main body of text to demonstrate that the results are robust to parameter variation.*

The sensitivity analyses have been referenced on lines 116-118 of the methods: “For further simulations with R_0=2.5 and R_0=3.5, together with a sensitivity analysis for other key parameters, please see §S1 of the Supplementary material.” and lines 344-346 of the discussion: “While our main results focus on a specific range of parameters, our findings are qualitatively robust to variations in these parameters as demonstrated by sensitivity analysis (§S1 of the Supplementary material).”

9. *It is not clear what the authors are suggesting with “Furthermore, behaviour change would likely have been exacerbated if healthcare services were overwhelmed” (line 280-281). Are the authors suggesting that the efficacy of RC interventions would increase if ICU capacity was overwhelmed? This needs further explanation.*

This has been reworded and explained more clearly on lines 301-303: “It is also likely that people would have increasingly limited their contacts if healthcare services were overwhelmed, which would have further reduced the likelihood of reaching the herd immunity threshold before shielding ended.”

10. *The authors state there would be “tens of thousands of avoidable deaths among those deemed to be lower risk” (line 286). “Avoidable” implies that these deaths are being compared to some baseline. This needs further explanation.*

This refers to the fact that in the best-case scenario (perfect shielding) there are no mitigations for those in the lower-risk category. This has now been made clear in the manuscript on lines 346-349: “Even in the best-case scenario with perfect shielding, our model estimates that there would have been tens of thousands of avoidable deaths among those deemed to be at lower risk due to limited mitigation in this subpopulation, even without accounting for the rapid depletion of healthcare capacity.”

11. *The statement "This effect is not seen in previous models which do not account for the clustering of higher risk members of the population", regarding reference (6) is false (lines 300-303) - as both models include population clustering based on risk status (the referenced model has a general, vulnerable and shielder population structure). Do the authors mean heterogeneity across subpopulations with regard to the probability of requiring ICU treatment (hospitalisation-based risk clustering)?*

This statement has been clarified as follows on lines 362-364: “This effect is not seen in previous models which do not differentiate between higher risk members of the population who reside in the community and those who reside in LTC facilities (6).”

12. *It would be beneficial to briefly explain what "shielding" is in the abstract – as this is only described in the introduction (line 14).*

A brief explanation has been added to the abstract stating that shielding means: “…(minimising their contacts with others)…”

13. *Figures 4 and 5 would be more readable with a standalone legend.*

We agree that this would be helpful for the readability. All relevant figures now have a key at the bottom to describe the four main line colours along with a text description.

14. *The probability of requiring ICU treatment given an individual is symptomatic is derived from Table S3 not Table S2 (line 209).*

We thank the reviewer for spotting this. It has now been amended.

15. *Please ensure that MATLAB code is accessible by Github users without a bath.ac.uk login.*

This link has been fixed so that anyone can access it.

Reviewer 2:

1. *You use an SEIR epidemic model and this seems to be overly simplistic given the existing knowledge about COVID-19 dynamics and characteristics. For example, by this simple SEIR formulation, you make no distinction between the asymptomatic and symptomatic infectious individuals. These categories respond differently to the disease (different hospitalization and mortality levels, yet these are key outputs in your study) and also contribute differently to the infection pressure with the symptomatic likely to shed more. Revise your model to further split I into symptomatic and asymptomatic as this will alter the dynamics significantly. You also miss the hospitalized compartment yet its output is among you key results. For an example on how these were captured, see Mugisha et al. 2021 PLoS ONE 16(2): e0247456.* [*https://doi.org/10.1371/journal.pone.0247456*](https://doi.org/10.1371/journal.pone.0247456) *and the cited literature therein)*

We appreciate that the dynamics of COVID-19 involve both symptomatic and asymptomatic cases, and one may choose to model these as distinct classes to capture differences in transmission, hospitalisation, and mortality. However, one may also group all infected individuals together and take the transmission and mortality parameters as ensemble averages over the infected class. This is a simpler approach and one which we believe is sufficient for the purposes of our study. It is also not clear that a symptomatic individual will necessarily have a higher transmission rate, as higher shedding may be counteracted by having a lower contact rate due to more obvious symptoms. We have verified that our assumption which groups symptomatic and asymptomatic cases together does not have a significant impact on the results, as demonstrated in the supplementary material (§S2, Fig. S15), where we have split the infectious class into symptomatic and asymptomatic classes, allowing for different transmission and mortality rates.

Similarly, we do not have a distinct class for hospitalisations, and instead assume simply that a proportion of infected individuals end up in hospital. We use the number of infectious in the population and the average ICU duration stay to estimate the ICU usage through time. Including a separate hospitalisation class would require significant structural changes to our model without offering any new qualitative insights. Our results demonstrate that in every shielding scenario, the number of ICU beds required greatly exceeds the surge capacity, often by an order of magnitude. The precise dynamics of hospitalisation will have minimal impact on this outcome: ICU beds would be rapidly and massively overwhelmed. It is true that hospitalisation may lead to a reduction in transmission to the general population, but substantial nosocomial can still occur. We have added some text to Section 2.3 to better explain our method (see lines 225-227): “These data allow us to calculate the probability that an individual who is infected requires ICU treatment. This probability is then a posteriori applied to our infection curve to estimate the numbers of people who would be in ICU.”

Of course, one could include heterogeneity in a variety of other ways, for example by varying adherence to NPIs or contact rates by age groups (e.g. POLYMOD data), but there is a cost in terms of complexity and the number of (potentially unknown) parameters required to capture additional heterogeneities. Given that splitting infections explicitly into symptomatic and asymptomatic classes does not qualitatively change our results, and adding a hospitalisation class is unlikely to qualitatively change our main results, we leave the model in the main text intact. We also note that many other studies of COVID-19 take a similar approach and do not capture the impact of symptomatics, asymptomatics, and hospitalisations (e.g. van Bunnik et al. 2021. Phil Trans R Soc B).

2. *You use and incubation period of 5 days and an infectious period of 2 days. In literature, there is a wide variation in these estimates and it better you explore a wider range for these and perhaps other parameters. Since you are working with a stochastic model, you could deploy a parameter sampling scheme in which the program picks a random number from a given distribution of the parameter at every iteration. The other option would be to perform a sensitivity analysis to assess the impact of these parameters on the study findings. This will improved credibility and reliance of your study findings. You attempt such an analysis by lowering R0 from 3 to 2.5 and also increasing it to 3.5; add further sensitivity analyses to this.*

Further sensitivity analyses for the incubation period and the infectious period have been included in the supplementary material (Figs. S8-S11). Our main results are robust to variation in these parameters.

3. *Given the existing knowledge, it is clear that suffering from the disease doesn’t confer permanent immunity. Therefore, you need amend you model to allow for waning of disease-induced immunity.*

We agree that waning immunity is an important consideration for the effectiveness of shielding strategies. We have therefore added this to our model as an additional scenario in the main text (Fig. 6). We group this result with external infections, as both are long-term effects that always make all shielding scenarios perform worse. We have added waning immunity to the schematic in Fig. 1 and the methods in equation (7e).

4. *In line 145, you write “In the imperfect and perfect shielding scenarios, shielding begins at the start of each simulation and ends once incidence falls below a threshold of 60 new cases per 100,000 in the population per week.” Justify your choice for this criteria.*

A line of text has been added to lines 158-159 stating that: “This threshold is chosen based on the number of new cases recorded in the UK on the 1st April 2021 when shielding advice ended (21).”

5. *Equation 8f on page 10 is wrong.*

We thank the reviewer for spotting this. It has now been corrected.

6. *The abstract needs to briefly mention the methods used.*

We have added “…using a stochastic SEIR framework…” to the abstract.

7. *Keywords are missing.*

Keywords have now been added.

8. *Line 33, change “lower-risk” to lower risk i.e. remove hyphen? Other occurrences of lower-risk are ok.*

This has been amended.

9. *Line 99, you write 75s, write this fully e.g. 75 year-olds for clarity.*

We have now updated this.

10. *Line 138, use “element-wise”?*

We have now changed this.

11. *Line 177, you simulate 600 days. Isn’t this timeframe long enough to warrant inclusion of the vital dynamics i.e. birth and nature deaths in the model?*

We do not include births and deaths as population turnover will always make shielding worse by eroding herd immunity. The dynamics would therefore be qualitatively similar to the waning immunity scenario but stretched out over a longer timescale. Including vital dynamics will not, therefore, alter our conclusions significantly. Again, the purpose of our study is to illustrate major problems with shielding strategies and population turnover would not fundamentally change any of our conclusions.
