# Supplementary material for: Critical weaknesses in shielding strategies for COVID-19
Source: PLOS Glob Public Health. 2022 Apr 26;2(4):e0000298. doi: 10.1371/journal.pgph.0000298 (PMC10021285; doi:10.1371/journal.pgph.0000298)
Supplement: Attachment — Submitted filename: Referee_comments.docx [file pgph.0000298.s003.docx]

**Department of Mathematical Sciences, University of Bath**

Claverton Down, Bath BA2 7AY, UK

February 28, 2022

Dear Editor,

Thank you for the reviews for our manuscript: “Critical weaknesses in shielding strategies for COVID-19”. We have carefully considered your comments and have revised our manuscript accordingly. We also thank reviewer 2 for taking the time to reassess our manuscript. Please find our detailed responses below, in which comments by the editor are *italicised* and our responses are in regular text underneath.

1. *L47: I think GBD influenced greatly these unfortunate decisions/thinking, rather than partly. Also, I would add, if authors agree, something on the lines of "while allowing uncontrolled viral transmission among 'low-risk' individuals" following focused protection of 'high-risk' individuals. Also, I suggest not citing their website as a way of avoiding potential spread of misinformation and dangerous approaches. Critiques or other type citations may be more appropriate perhaps?
   Examples (there may be others preferred by the authors):* [*https://doi.org/10.1136/bmj.m3908*](https://eur01.safelinks.protection.outlook.com/?url=https%3A%2F%2Fdoi.org%2F10.1136%2Fbmj.m3908&data=04%7C01%7Ccs640%40bath.ac.uk%7Ce941320bda5d44afff8f08d9f877eaa4%7C377e3d224ea1422db0ad8fcc89406b9e%7C0%7C0%7C637814016178525680%7CUnknown%7CTWFpbGZsb3d8eyJWIjoiMC4wLjAwMDAiLCJQIjoiV2luMzIiLCJBTiI6Ik1haWwiLCJXVCI6Mn0%3D%7C3000&sdata=sYI3QuSLodpmAFCBflyRtvqDzI11brF78FIbAyE4Oxw%3D&reserved=0) [*https://theconversation.com/5-failings-of-the-great-barrington-declarations-dangerous-plan-for-covid-19-natural-herd-immunity-148975*](https://eur01.safelinks.protection.outlook.com/?url=https%3A%2F%2Ftheconversation.com%2F5-failings-of-the-great-barrington-declarations-dangerous-plan-for-covid-19-natural-herd-immunity-148975&data=04%7C01%7Ccs640%40bath.ac.uk%7Ce941320bda5d44afff8f08d9f877eaa4%7C377e3d224ea1422db0ad8fcc89406b9e%7C0%7C0%7C637814016178525680%7CUnknown%7CTWFpbGZsb3d8eyJWIjoiMC4wLjAwMDAiLCJQIjoiV2luMzIiLCJBTiI6Ik1haWwiLCJXVCI6Mn0%3D%7C3000&sdata=Jw4RreRT6X71S%2FN7zL37lAYT%2Bspob1jRYR%2FigzmkQqg%3D&reserved=0) [*https://doi.org/10.1186/s12879-021-06357-4*](https://eur01.safelinks.protection.outlook.com/?url=https%3A%2F%2Fdoi.org%2F10.1186%2Fs12879-021-06357-4&data=04%7C01%7Ccs640%40bath.ac.uk%7Ce941320bda5d44afff8f08d9f877eaa4%7C377e3d224ea1422db0ad8fcc89406b9e%7C0%7C0%7C637814016178525680%7CUnknown%7CTWFpbGZsb3d8eyJWIjoiMC4wLjAwMDAiLCJQIjoiV2luMzIiLCJBTiI6Ik1haWwiLCJXVCI6Mn0%3D%7C3000&sdata=oT6J4ICIjq2NHI%2F4tubYxV%2FBz3QUu0Cjni2uZUcTqlU%3D&reserved=0)

We have reworded a couple of the sentences in the introduction as per your suggestions, stressing that the GBD essentially called for the widespread infection of lower-risk individuals. We have also removed the reference to the GBD website and replaced it with two of those you have suggested.

2. *L66: Check this line which is incomplete "(see §2.1 and )”*

This has been fixed.

3. *L123: Change "infection with COVID-19" to "SARS-CoV-2 infection"*

Corrected as requested.

4. *L308 and L345: Here is the same case as in L66: "descriptions as in" ? The clean version in these three cases seems to drop an error: "Error! Reference source not found." Pleasure ensure citations field codes are not corrupted and citations are accurate throughout the document.*

Thank you for bringing these to our attention. We have corrected these instances and checked every other instance where this corruption could have occurred.

5. *Keywords: These should be consistent between the main text and its supplementary material since it's the same manuscript.*

These now match.
